# Application of therapeutic plasma exchange in dogs with immune‐mediated thrombocytopenia

**DOI:** 10.1111/jvim.15836

**Published:** 2020-06-19

**Authors:** Lucy Kopecny, Carrie A. Palm, Sean Naylor, John Kirby, Larry D. Cowgill

**Affiliations:** ^1^ Department of Veterinary Medicine and Epidemiology University of California, Davis Davis California USA; ^2^ William R. Pritchard Veterinary Medical Teaching Hospital, University of California, Davis Davis California USA

**Keywords:** apheresis, autoimmune, canine, platelet

## Abstract

Therapeutic plasma exchange (TPE) is an emerging treatment for dogs with immune‐mediated diseases, but reports for treatment of immune‐mediated thrombocytopenia (IMT) are lacking. These case reports illustrate the application of centrifugal TPE in 4 dogs with IMT. All dogs presented with severe hemorrhage requiring ≥1 blood transfusions, were unresponsive to conventional treatment or both. Dogs were treated with 3 sequential centrifugal TPE sessions, totaling 4.0 to 4.9 total plasma volumes exchanged per dog. In 3 dogs, TPE was associated with improvement in clinical manifestations of bleeding and platelet count in combination with immunosuppressive drugs. One dog was euthanized after 3 treatments because of persistent severe thrombocytopenia and hemorrhage. Preliminary observations indicate that TPE is safe and may be a useful adjunct in the management of IMT that is severe or refractory to traditional treatment.

**Abbreviations**
ACD‐A: anticoagulant citrate dextrose solution
BUN: blood urea nitrogen
dexSP: dexamethasone sodium phosphate
FFP: fresh frozen plasma
HCT: hematocrit
hIVIG: human IV immunoglobulin
hpf: high power field
IMHA: immune‐mediated hemolytic anemia
IMT: immune‐mediated thrombocytopenia
pRBC: packed red blood cell
RBC: red blood cell
TBV: total blood volume
TPE: therapeutic plasma exchange

## 1 INTRODUCTION

Immune‐mediated thrombocytopenia (IMT) is a disease in which antibodies bind to platelet surface epitopes and lead to platelet destruction.^1^, ^2^ Resulting thrombocytopenia is severe and risk for spontaneous hemorrhage often develops when platelet counts are <50 000/μL.^3^ Immune‐mediated hemolytic anemia (IMHA) is diagnosed concurrently in some cases, a condition known as Evan’s syndrome.^4^

Treatment for dogs with primary IMT consists of immunosuppression with corticosteroids, alone, or in combination with other immunosuppressive drugs including azathioprine, cyclosporine, and mycophenolate mofetil.^5^, ^6^, ^7^ Vincristine and human IV immunoglobulin (hIVIG) decrease the time required to restore platelet counts to ≥40 000/μL in affected dogs.^8^, ^9^, ^10^ Splenectomy also has been used in dogs with refractory IMT.^11^

Therapeutic plasma exchange (TPE) is an extracorporeal treatment in which a patient’s plasma, containing pathogenic substances such as antibodies and antigen‐antibody complexes, is removed and exchanged with replacement solutions.^12^ In dogs, TPE is emerging as an effective treatment for immune‐mediated disorders, including IMHA and myasthenia gravis, but its application in dogs with IMT has not been described previously.^13^, ^14^, ^15^, ^16^ With these case reports, we aimed to describe the techniques, complications, and outcomes of TPE in 4 dogs treated for IMT.

## 2 MATERIALS AND METHODS

The medical records of 4 dogs that underwent TPE between 2016 and 2018 at the William R. Pritchard Veterinary Medical Teaching Hospital, University of California, Davis (UC Davis VMTH) for treatment of IMT were reviewed. Criteria for initiation of TPE were unresponsiveness to ≥4 days of immunosuppressive treatment and ongoing hemorrhage with need for ≥1 packed red blood cell (pRBC) transfusion. Informed client consent was obtained before starting TPE.

Clinical evaluation in all dogs included thoracic radiographs, abdominal ultrasound examination, and qualitative testing for antibodies against *Anaplasma* spp., *Ehrlichia* spp., and *Borrelia burgdorferi* and heartworm antigen (IDEXX SNAP 4Dx, IDEXX Laboratories, Inc, Westbrook, Maine). Additional testing is noted in the individual case descriptions. Time to platelet count ≥40 000/μL was recorded.

In all dogs, a double‐lumen catheter was placed percutaneously in an external jugular vein using the modified Seldinger technique^17^ under sedation or general anesthesia and was maintained throughout hospitalization. No complications occurred during catheter placement.

Three sequential centrifugal TPE sessions were performed in each dog using the Spectra Optia (Terumo BCT, Lakewood, Colorado) apheresis system. Therapeutic plasma exchange was performed on day 0, 1, and then on days 3 or 4 for all dogs. For each TPE session, exchange of 1.5 plasma volumes was prescribed to achieve an approximate 80% decrease in antibody burden.^18^ Plasma volume (liters) was calculated according to the formula: (1‐hematocrit [HCT]) × (body weight [kg] × 0.08). The extracorporeal circuit (approximately 141 mL) was primed initially with 0.9% NaCl. In some treatments, the circuit was reprimed with variable volumes of 0.9% NaCl in combination with pRBC and fresh frozen plasma (FFP). This secondary priming was performed to decrease the risk of hypovolemia, hypotension, hemodilution, or some combination of these related to small total blood volume (TBV) relative to extracorporeal circuit size and to achieve PCV >20% at treatment initiation. Replacement fluids included combinations of 6% hetastarch, 0.9% NaCl, FFP, and pRBC, and were individualized based on TBV, PCV, and plasma fibrinogen concentration. Administration of 6% hetastarch was limited to <15 mL/kg per treatment (with the majority of replacement hetastarch removed along with waste plasma). Percentage of FFP replacement used was dependent on pretreatment plasma fibrinogen concentration and number of consecutive treatments with associated risk of fibrinogen and clotting factor depletion. Regional anticoagulation with citrate dextrose solution (ACD‐A) was used in all cases according to the procedures established in the Spectra Optia operating system. Calcium gluconate 10% (0.5‐2 mL/kg/h) was supplemented IV during TPE treatments to maintain serum ionized calcium concentration >0.9 mmol/L. Systemic heparinization was utilized during selected treatments because of concerns for extracorporeal thrombosis. Diphenhydramine hydrochloride (2 mg/kg SC) was administered 30 minutes before TPE initiation to decrease risk of a type I hypersensitivity reaction to blood products.

Heart rate, respiratory rate, temperature, blood pressure, HCT, and serum ionized calcium concentration were recorded every 15 to 30 minutes. Pulse oximetry and electrocardiography were monitored as indicated. Activated clotting time (ACT) was measured in any dog systemically heparinized (Medtronic ACT II Monitor, Dublin, Ireland). All adverse reactions were recorded and described in individual case histories.

## 3 CASE HISTORY

### 3.1 Case 1

#### 3.1.1 Clinical features

A 5.8 kg, 4‐year‐old female spayed Dachshund (dog 1) was evaluated for gingival hemorrhage and hyporexia. Initial physical examination identified cutaneous petechiae and ecchymoses. Both melena and hematochezia were present.

An initial CBC disclosed a regenerative anemia (HCT, 34.3%; reference range [RR], 40%‐55%; reticulocytes, 279 900/μL; RR, 7000‐65 000), neutrophilia (12 773/μL; RR, 3000‐10 500/μL) with left shift (bands, 788/μL; RR, rare), and thrombocytopenia (5000/μL; RR, 150‐400 000/μL) with increased MPV (13.8 fl; RR, 7‐13 fl). Hyperbilirubinemia (1.4 mg/dL; RR, 0‐0.2 mg/dL), hyperglycemia (127 mg/dL; RR, 86‐118 mg/dL) and hypokalemia (3.0 mmol/L; RR, 3.6‐4.8 mmol/L) were present. Urinalysis of a voided sample disclosed hematuria (>100 RBC/high power field [hpf]; RR, 0‐2/hpf). Bacterial urine culture was negative. Echocardiography was normal. Direct Coomb’s test was weakly positive at 1:4 dilution. Direct slide agglutination test was negative and no spherocytes were present before blood transfusion. Immediately before TPE, the dog had progressive regenerative anemia (HCT, 18.7%; reticulocytes, 312 300/μL).

Dog 1 was diagnosed with primary IMT, with Evan’s syndrome considered less likely given equivocal results.

#### 3.1.2 Treatment

Immunosuppressive treatment before presentation consisted of prednisolone (0.9 mg/kg PO q12h) and cyclosporine (8.6 mg/kg PO q12h) at the referring veterinarian for 3 days, transitioned to dexamethasone sodium phosphate (dexSP; 0.2 mg/kg/day IV) and cyclosporine (5 mg/kg IV q24h) on admission to UC Davis VMTH for 3 additional days before TPE. There was no response to 6 days of these treatments. Three platelet transfusions were administered because of severe hemorrhage.

**FIGURE 1:**
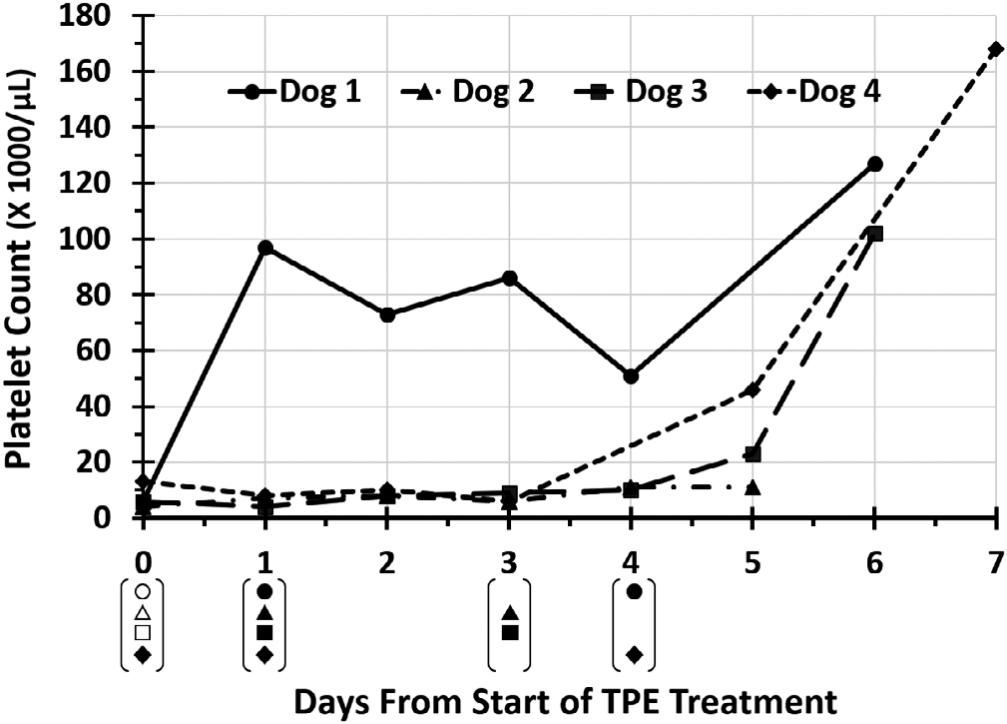
Change in platelet counts from day 0 (first day of therapeutic plasma exchange [TPE]) to day 7 in 4 dogs with immune‐mediated thrombocytopenia (IMT). Symbols in brackets underneath days from start of TPE treatment indicate days on which each dog received TPE treatments. Open symbols in brackets indicate that the dog received a platelet transfusion. Platelet count for dog 4 on day 4 was unable to be determined as platelets were clumped

Dog 1 received 3 TPE treatments over 5 days for a total of 4.0 plasma volumes (Figure 1). Treatment variables for TPE are summarized in Table 1. Hematologic data on the day of starting TPE is provided in Supplemental Table 1 for all dogs.

**TABLE 1:** Treatment parameters during 12 TPE sessions in 4 dogs with median (range)

	Median (range)
Treatment time (minutes)	242 (144‐302)
Calculated patient plasma volume (mL)	382 (170‐1208)
Percentage of replacement solution as fresh frozen plasma (%) per TPE session	100 (40‐100)
Percent patient blood volume in extracorporeal circuit (%)	27.4 (10‐66)
Plasma removed (mL)	544 (324‐1637)
Plasma volumes exchanged per TPE session	1.4 (1‐1.8)
Total plasma volumes exchanged per dog over all treatments	4.4 (4.0‐3.9)
Packed cell volume pre‐TPE (%)	20 (17‐32)
Serum ionized calcium concentration pre‐TPE (mEq/L; RR, 1.3‐1.46 mEq/L)	1.28 (1.18‐1.32)
Serum ionized calcium concentration post‐TPE (mEq/L; RR, 1.3‐1.46 mEq/L)	1.24 (1‐1.5)

Abbreviations: RR, reference range; TPE, therapeutic plasma exchange.

Clinical signs suggestive of hypovolemia were responsive to bolus infusion of 0.9% NaCl during the third TPE session. No other TPE‐related complications were noted.

#### 3.1.3 Outcome

**FIGURE 2:**
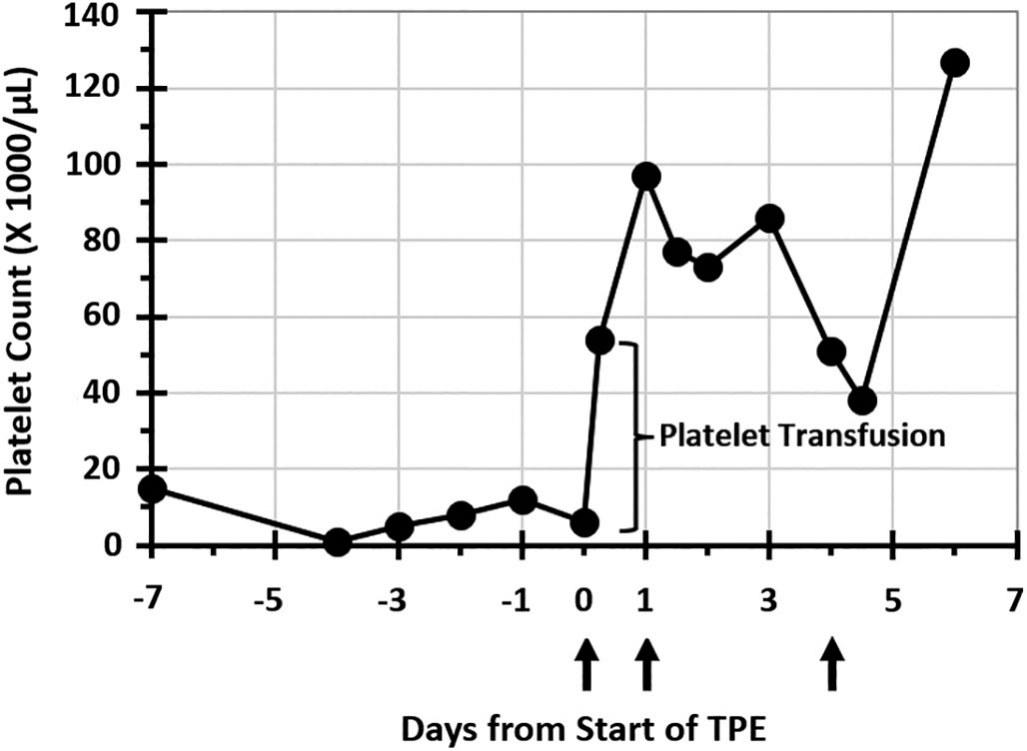
Changes in platelet counts for dog 1 in relation to days from presentation, including platelet counts pre and post‐therapeutic plasma exchange (TPE) on days 0, 1, and 4. Day 0 is first day of TPE. Upward facing arrows indicate days on which dog 1 underwent TPE treatments

Dog 1 was hospitalized for 7 days, including 4 days after starting TPE. Changes in platelet count in relation to TPE treatments for all dogs are presented in Figure 1. More detailed data of changes in platelet count in dog 1 in relation to TPE sessions and platelet transfusions are shown in Figure 2. Time to platelet count ≥40 000/μL was 7 days after starting immunosuppressive treatment and 1 day after starting TPE. Six days after starting TPE, platelet count was 127 000/μL with improved regenerative anemia (HCT, 35.0%; reticulocytes, 150 900/μL) and resolution of hyperbilirubinemia (0.2 mg/dL). Melena and hematochezia resolved 1 day after starting TPE.

Dog 1 was discharged on prednisolone (0.9 mg/kg PO q12h) and cyclosporine (Atopica, Elanco Inc, Greenfield, Indiana; 5.2 mg/kg PO q12h). The dog was euthanized 44 days after discharge because of weight loss, hyporexia, and vomiting considered unrelated to IMT. Immune‐mediated thrombocytopenia was controlled at the time of euthanasia.

### 3.2 Case 2

#### 3.2.1 Clinical features

A 16.8 kg, 10‐year‐old female spayed Beagle (dog 2) was evaluated for a 3‐day history of hematochezia after a prophylactic rattlesnake vaccine administered 4 days before onset of clinical signs. Initial physical examination disclosed obtundation, tachycardia (170 beats per minute), tachypnea (36 breaths per minute), and a grade II/VI left apical systolic heart murmur. Gingival hemorrhage, cutaneous petechiae, and ecchymoses and melena were present.

The initial CBC showed regenerative anemia (HCT, 11.7%; reticulocytes, 134 600/μL), neutrophilia (11 448/μL) with left shift (bands, 322/μL), monocytosis (3225/μL; RR, 150‐1200/μL), and thrombocytopenia (8000/μL). No spherocytes were present. A biochemistry profile disclosed increased blood urea nitrogen (BUN) concentration (47 mg/dL), hypoalbuminemia (1.9 g/dL; RR, 3.4‐4.3 g/dL), hyperglycemia (193 mg/dL), hypocholesterolemia (84 mg/dL; RR, 139‐353 mg/dL), hypokalemia (2.1 mmol/L), and hyponatremia (136 mmol/L; RR, 143‐151 mmol/L). Serum bilirubin concentration was normal (0.2 mg/dL). Urinalysis on a voided sample disclosed hematuria (50‐100 RBC/hpf). An echocardiogram showed no evidence of endocarditis. An electrocardiogram identified a frequent accelerated idioventricular rhythm and occasional ventricular premature beats. The dog was diagnosed with IMT, suspected to be primary or possibly triggered by the prophylactic rattlesnake vaccine. Disseminated intravascular coagulation was considered less likely because there was no evidence of thrombosis, schistocytosis, and plasma fibrinogen concentration was normal in this dog.

#### 3.2.2 Treatment

Therapeutic plasma exchange was initiated 4 days after starting immunosuppressive medications (dexSP [0.2 mg/kg q24h] and cyclosporine [5 mg/kg q24h] IV). Three platelet transfusions were administered 2 days before initiating TPE because of ongoing severe hemorrhage.

Dog 2 received 3 TPE treatments over 4 days, providing a total of 4.0 plasma volumes exchanged over all treatments.

Clinical signs suggestive of hypovolemia and responsive to bolus infusions of FFP occurred during the first TPE session. The third TPE session was temporarily discontinued while the extracorporeal circuit was replaced because of severe clotting in the extracorporeal catheter and inlet line before ACD‐A infusion that obstructed the downstream inlet blood filter.

#### 3.2.3 Outcome

Dog 2 was hospitalized for 8 days and euthanized 2 days after the third TPE treatment because of persistent thrombocytopenia (11 000/μL) and hemorrhage requiring ongoing blood transfusions; there was no response to treatment. This dog required 22 pRBC units during hospitalization.

### 3.3 Case 3

#### 3.3.1 Clinical features

A 2.8 kg, 4.6‐year‐old female spayed terrier mixed breed dog was presented for hematemesis. Physical examination identified cutaneous petechiae and ecchymoses, gingival hemorrhage, and hematochezia on rectal examination. Vital signs were normal on presentation.

Admission CBC disclosed a regenerative anemia (HCT, 39.7%; reticulocytes, 121 100/μL), left shift (bands, 1844/μL), monocytosis (1721/μL), and thrombocytopenia (6000/μL) with increased MPV (17.3 fl). Serum biochemistry profile disclosed increased BUN concentration (51 mg/dL), hypoalbuminemia (2.4 g/dL), hyperglycemia (152 mg/dL), hypocholesterolemia (102 mg/dL), and hypokalemia (3.5 mmol/L). Urinalysis on a voided sample was normal. An echocardiogram was normal. Aerobic bacterial blood cultures were negative. Quantitative PCR testing was negative for *Anaplasma* spp., *Bartonella* spp., *B. burgdorferi*, *Ehrlichia canis*, *Rickettsia* spp., *Babesia* spp., and *Mycoplasma haemocanis*. Primary IMT was diagnosed.

#### 3.3.2 Treatment

Initial immunosuppressive treatment consisted of dexSP (0.2 mg/kg/day IV). Cyclosporine (3.6 mg/kg PO q12h) was started on the first day of TPE. A single platelet transfusion was administered on day 0 before TPE to decrease the risk of hemorrhage during catheter placement because dog 3 previously had developed marked hematomas at venipuncture sites. Therapeutic plasma exchange was initiated after 4 days of immunosuppressive treatment because of severe ongoing hemorrhage requiring 2 pRBC transfusions.

Dog 3 received 3 TPE treatments over 4 days, providing 4.8 plasma volumes of cumulative treatment (Figure 1; Table 1).

Vomiting and hyperthermia (from 100.5 °F to 102.3 °F), suspected to represent a nonhemolytic febrile transfusion reaction, occurred during the third TPE session; treatment discontinuation was not necessary. Extracorporeal circuit clotting occurred during the third TPE session but did not necessitate treatment discontinuation.

#### 3.3.3 Outcome

Dog 3 was hospitalized for 11 days, including 6 days after TPE initiation. Time to platelet count ≥40 000/μL was 10 days after starting immunosuppressive treatment and 5 days after starting TPE. Hematochezia resolved 4 days after starting TPE. Platelet count was 453 000/μL 12 days after starting immunosuppressive treatment and 8 days after TPE initiation.

Dog 3 was discharged on prednisone (0.9 mg/kg PO q12h) and cyclosporine (3.6 mg/kg PO q12h) and was in remission at last follow‐up (417 days), with platelet count 348 000/μL 6 months after discontinuing immunosuppression.

### 3.4 Case 4

#### 3.4.1 Clinical features

An 8.3 kg, 6‐year‐old male castrated terrier mixed breed dog (dog 4) was presented for evaluation of uncontrolled IMT. The dog presented to the referring veterinarian for evaluation of cutaneous petechiae and ecchymoses, with a platelet count of 18 000/μL. The dog was immunosuppressed for 8 days before presentation using prednisolone 0.9 mg/kg PO q12h and cyclosporine 6 mg/kg PO q24h and had received 3 pRBC transfusions because of progressive anemia (PCV, 9%‐15% before transfusion). Physical examination at admission identified a grade III/VI left apical systolic heart murmur, cutaneous petechiae and ecchymoses, and melena.

An initial CBC disclosed regenerative anemia (HCT, 26.4%; reticulocytes, 188 200/μL), neutrophilia (11 146/μL) with left shift (bands, 1911/μL), monocytosis (1911/μL), and thrombocytopenia (7000/μL) with increased MPV (14.9 fl). A serum biochemistry profile disclosed hypoalbuminemia (2.1 g/dL), hypoglobulinemia (1.6 g/dL; RR, 1.7‐3.1 g/dL), hyperglycemia (132 mg/dL), increased ALP activity (135I U/L; RR, 14‐91 IU/L), hypocholesterolemia (125 mg/dL), and hyperbilirubinemia (0.3 mg/dL) after pRBC transfusions. Urinalysis on a voided sample was normal. Urine culture and aerobic and anaerobic blood cultures were negative. Primary IMT was diagnosed.

#### 3.4.2 Treatment

Immunosuppressive treatment was changed to dexSP (0.3 mg/kg IV q24h) and cyclosporine (5 mg/kg IV q24h) at admission.

Overall, 4.9 plasma volumes were exchanged during 3 TPE sessions over 5 days.

During the second TPE session, severe clotting occurred in the extracorporeal circuit interrupting treatment until the extracorporeal circuit was replaced. Similar to dog 2, clotting occurred in the extracorporeal catheter and inlet line before ACD‐A infusion, likely because of a low inlet blood flow rate of 2.8 mL/min. On the subsequent TPE session, a continuous rate infusion of heparin was administered to maintain ACT between 120 and 140 seconds (RR, 80‐120 seconds). Despite heparin infusion, a clot occurred in the inlet line, but did not interrupt treatment because no change in inlet pressures occurred.

#### 3.4.3 Outcome

Dog 4 was hospitalized for 7 days, including 5 days after TPE initiation. Time to platelet count ≥40 000/μL was 15 days after starting immunosuppressive treatment and 5 days after starting TPE. Platelet count normalized 17 days after starting immunosuppressive treatment and 7 days after starting TPE (platelet count, 168 000/μL). Melena improved within 2 days of starting TPE; platelet count at this time was 10 000/μL with clumping.

Dog 4 was discharged on prednisone (0.9 mg/kg PO q12h) and cyclosporine (4.2 mg/kg PO q12h) and was in remission at last follow‐up (87 days postdischarge) with a platelet count of 363 000/μL.

## 4 DISCUSSION

This case series represents the first report of the application of TPE in dogs for treatment of IMT. Centrifugal TPE was safe in small and medium‐sized dogs with IMT, and when combined with immunosuppression, was associated with improved platelet counts and resolution of clinical bleeding in 3/4 dogs. During TPE, circulating pathogenic substances including antibodies and antibody‐antigen complexes are removed in waste plasma.^12^, ^19^ Antiplatelet antibodies are recognized in dogs with IMT^20^, ^21^ and can cause platelet dysfunction.^22^ Thus, it is conceivable that TPE could decrease ongoing platelet destruction associated with antiplatelet antibodies and contribute to improved platelet function. This hypothesis has formed the basis for its use in humans, although evidence is limited.^19^

All dogs in this case series were treated concurrently with TPE and immunosuppressive drugs and our results did not distinguish between the effects of these treatments and were not compared to a control group. Consequently, specific benefits of TPE on IMT control cannot be conclusively assessed. However, case selection was biased strongly toward dogs with severe clinical manifestations of bleeding requiring multiple blood transfusions, and not rapidly responsive to traditional medical management. Negative prognostic indicators of increased BUN concentration, melena, or both at admission were present in 3/4 dogs. Survival in dogs with melena or increased BUN at admission has been reported to be 60.0 and 57.1%, respectively.^5^

A priority of IMT is to rapidly decrease life‐threatening bleeding by increasing the number of functional platelets. In previous retrospective studies evaluating prednisone, with or without other immunosuppressive agents, a median of 10 days was required to establish a platelet count >150 000/μL^23^ and 7.1 days to reach 100 000 platelets/μL.^7^ Other adjunctive treatments such as vincristine and hIVIG are reported to increase platelet counts more rapidly than standard immunosuppressive treatments.^8^, ^9^, ^10^ Mean time to platelet count ≥40 000/μL was 4.9 (±1.1) days in dogs treated with prednisone and vincristine and 6.8 (±4.5) days in dogs treated with prednisone alone^9^ and median time to platelet count ≥40 000/μL was 2.5 days in dogs treated with vincristine or hIVIG in combination with prednisone.^8^ In our case series, 1 to 6 days were required to reach a platelet count ≥40 000/μL after TPE. This time is similar to that of other adjunctive treatments, but comparative benefits of other therapeutic strategies remain to be defined with future comparative studies in dogs with similar disease severity.

Although application of TPE is limited by substantial expense and availability, more rapid control of active hemorrhage may lower overall hospital costs. Other adjunctive treatments such as hIVIG and vincristine also have potential limitations and as such are rarely utilized at our institution. An in vitro study suggested that vincristine inhibits platelet aggregation in dogs and raised concerns its use in IMT might result in impaired platelet function despite an increased platelet count,^24^ but this outcome has not been reported clinically.^8^ Vincristine also lacks specific immunomodulatory effects. A disadvantage of hIVIG, in addition to cost, is its potential for adverse effects associated with exposure to a heterologous protein of human origin, although several studies have shown no immediate or delayed adverse effects.^8^, ^10^, ^25^

The small number of dogs and lack of a comparator group not receiving TPE are limitations of this case series. Variations in treatment protocol, including time to initiation of TPE and TPE schedule, may have influenced platelet recovery. Based on our case series, prospective studies that include a control group and adjust for confounders such as disease severity are warranted.

## 5 CONCLUSIONS

In dogs with IMT, TPE was safe and associated with improved platelet counts and clinical manifestations of bleeding in 3/4 dogs treated concurrently with immunosuppression. It has a rational basis for interfering with the pathogenesis of IMT and the potential to alter the immediate, life‐threatening clinical manifestations of IMT. Additional studies are needed to fully define the role of TPE in this potentially life‐threatening disease.

## CONFLICT OF INTEREST DECLARATION

Authors declare no conflict of interest.

## OFF‐LABEL ANTIMICROBIAL DECLARATION

Authors declare no off‐label use of antimicrobials.

## INSTITUTIONAL ANIMAL CARE AND USE COMMITTEE (IACUC) OR OTHER APPROVAL DECLARATION

Authors declare no IACUC or other approval was needed.

## HUMAN ETHICS APPROVAL DECLARATION

Authors declare human ethics approval was not needed for this study.

## Supporting information


**Supplemental Table 1** Summary of hematologic data for four dogs with IMT on the day of starting TPE.
