# Supplementary material for: Application of therapeutic plasma exchange in dogs with immune‐mediated thrombocytopenia
Source: J Vet Intern Med. 2020 Jun 19;34(4):1576–81. doi: 10.1111/jvim.15836 (PMC7379011; doi:10.1111/jvim.15836)
Supplement: Supplementary file 1 — Supplemental Table 1 Summary of hematologic data for four dogs with IMT on the day of starting TPE. [file JVIM-34-1576-s001.pdf]

**Supplemental Table 1.** Summary of hematologic data for four dogs with IMT on the day of starting TPE.

| | **Reference range** | **Dog 1** | **Dog 2** | **Dog 3** | **Dog 4** |
|---|---|---|---|---|---|
| HCT (%) | 40-55 | 18.7 | 18.9 | 19.2 | 23.6 |
| Reticulocyte count (/µL) | 7,000-65,000 | 312,300 | - | 279,000 | 396,000 |
| White blood cell count (/µL) | 6,000-13,000 | 16,865 | 37,070 | 25,417 | 17,760 |
| Neutrophil count (/µL) | 3,000-10,500 | 12,143 | - | 17,538 | 14,030 |
| Band neutrophils (/µL) | Rare | 2,530 | - | 3,813 | 1,243 |
| Platelet count (/µL) | 150-400,000 | 6,000 | 17,000 | 6,000 | 13,000 |
| Mean platelet volume (fl) | 7-13 | 12.3 | 13.7 | 16.0 | 12.2 |
| Plasma protein (g/dL) | 6.0-8.0 | 4.8 | - | 5.4 | 4.6 |
